# Temporal organization skills in cochlear implants recipients

**DOI:** 10.1016/S1808-8694(15)30149-X

**Published:** 2015-10-18

**Authors:** Patrícia Danieli Campos, Kátia de Freitas Alvarenga, Natália Barreto Frederigue, Leandra Tabanez do Nascimento, Koichi Sameshima, Orozimbo Alves Costa Filho, Maria Cecília Bevilacqua

**Affiliations:** a Specialist in sound amplification lab - Hospital de Reabilitação de Anomalias Craniofaciais da Universidade de São Paulo, HRAC/USP, Bauru/SP; b PhD, Professor - Department of Speech and Hearing Therapy - Faculdade de Odontologia de Bauru, Universidade de São Paulo, FOB/USP; c PhD, Speech and Hearing therapist - Centro de Pesquisas Audiológicas do Hospital de Reabilitação de Anomalias Craniofaciais da Universidade de São Paulo, HRAC/USP; d PhD, Speech and Hearing therapist - Centro de Pesquisas Audiológicas do Hospital de Reabilitação de Anomalias Craniofaciais da Universidade de São Paulo, HRAC/USP, Bauru/SP; e Associate Professor - Department of Radiology and Cognitive Neurosciences Lab - LIM-43 University of São Paulo Medical School, São Paulo/SP; f Associate Professor, Cochlear Implant Program Coordinator - CPA do HRAC/USP, Bauru/SP; g Associate Professor, Full Professor and Head of the Speech and Hearing Department FOB/USP, Bauru/SP; h Hospital de Reabilitação de Anomalias Craniofaciais da Universidade de São Paulo

**Keywords:** cochlear implantation, auditory perception, hearing loss, hearing disorders

## Abstract

Processing acoustic clues from the sounds of speech depends on the proper perception of the frequency and duration of stimuli as a sequence of events. **Aim**: To assess the capacity for temporal organization in users of multichannel CI. **Method**: 14 normal hearing individuals formed the control group, matching in age and gender other 14 users of multichannel CI, who made up the study group, and they were assessed and compared as to the Frequency Patterns Test (FPT) and Duration Patterns Test (DPT). **Results**: CI users had good performance in temporal organization tasks, with mean results of 48.7% in the FPT and 59.6% in the DPT. For the control group, mean performance at the FPT was of 63.4% and in the DPT of 64.6%. We did not see statistically significant difference between the results from the control and study groups. **Conclusion**: the CI provided favorable performance in the tasks that required temporal organization skill for individuals evaluated in this study.

## INTRODUCTION

Auditory processing involves the behavioral phenomena of sound lateralization and location, auditory discrimination, recognition of auditory patterns, temporal aspects of hearing (temporal resolution, temporal masking, temporal integration and temporal ordering), auditory perception of a competitive acoustic sign and distorted acoustic signs^1^.

Auditory processing starts as of the sound capture by the external acoustic meatus all the way to the time when the acoustic event is experienced by the listener. The acoustic sign is filtered, transduced, codified, decoded and processed along the auditory system, until eventually it is perceived by the individual. Thus, we can say that the auditory perception is the result of auditory signal processing^2^.

The behavioral evaluation of the temporal auditory processing aims at analyzing individual performance as to their capacity to detect and discriminate stimuli variations along time^3^. The global objective of studies that involve temporal sequencing and ordering tasks is to determine the competence through which a person identifies these stimuli and locates the neural structures involved or committed with this skill.^4^

Two temporal sequencing detection and identification tests very much used today are the Pitch Pattern Sequence Test (PPST) and the Duration Pattern Sequence Test (DPST). Technically, such tests can be applied in the open field, since normative studies did not show significant differences between right and left ears^5^, ^6^.

In a study involving children from seven years of age all the way to 11 years and 9 months, Balen compared PPST and DPST results among females and males by age range, and did not notice statistically significant differences7 between males and females when compared in PPST and DPST. ^8^

Individuals with hearing impairment presented a loss in sound sensation that allows for the discrimination between low/high, strong/weak and long/short sounds^9^. Sensorineural hearing loss distorts the sound resulting in sensitivity reduction, abnormal increase in the sensation of intensity, decline in frequency selectiveness and a reduction in temporal resolution. With the involvement of the frequency resolution capacity, there is difficulty in speech perception, especially when we consider competitive noise. Speech temporal envelope, which codifies information is distorted in an altered auditory system, resulting in speech perception distortions^10^.

Individuals with hearing deficiency have more difficulties than people with normal hearing in temporal sequencing and ordering because of perception deficits^11^. Nonetheless, other studies reported that PPST and DPST seem to resist cochlear alterations^8^, ^12^, ^13^.

One of the therapeutic resources for profound and severe sensorineural hearing impairment (re) habilitation is the cochlear implant (CI), or bionic ear, an electronic device which replaces the organ of Corti and directly stimulates the ganglion cells of the auditory nerve, giving the individual the sensation of hearing.

The CI is made up of internal - surgically inserted, and external components, which include the antenna, transmission wires and speech processor. The latter continuously analyzes the speech sound and those of the environment and codifies them, preserving the important characteristics of speech temporal information and spectrum. Information from the acoustic signal spectrum is codified by the stimulation of different electrodes and the temporal information is codified by the temporal control of auditory nerve fiber firings^14^, ^15^, ^16^, ^17^.

The CI decodes the sound pattern in frequencies and intensities by means of electrical stimuli in electrodes located in different portions of the cochlea, thus leading to speech recognition. Nonetheless, sound decoding is not easily reproduced^18^.

The performance level in speech perception reached by individuals with CI is directly associated to the speed in which information can be processed, electronically transmitted and decoded successfully by the nervous system^19^.

The goal of the present investigation was to carry out a preliminary investigative study about the temporal ordering capacity of users of multichannel CI.

## METHODS

This study was approved by the Ethics in Research Committee under protocol # 148/2007-SVAPEPE-CEP. All the participants signed a Free and Informed Consent Form.

The evaluation protocol was made up of two behavioral tests, the Pitch Pattern Sequence Test (PPST) and the Duration Pattern Sequence Test (DPST). We used the comparative methodology of control and experimental groups.

## MATERIALS

The experimental group was made up of individuals using multichannel CI, older than 8 years of age at the time of the tests, who had been effectively using the CI for at least 6 months, complete electrode insertion in the cochlea and speech recognition in an open setting.

The control group was made up of normal hearing individuals, checked by means of threshold tonal audiometry, speech audiometry and tympanometry, ipsilateral and contralateral stapes reflex, matching the experimental group in age and gender. As inclusion criteria, the individuals could not have a prior history of pre or perinatal complications, neuro-psychomotor development delays, language development delays, past of repetition otitis, difficulties to read and write, musical knowledge, articulatory alteration or hyperactivity/attention deficit. This information was obtained by means of a questionnaire held before the tests.

The patients were then divided in two groups:

• Experimental group: 14 individuals using multichannel CI, 7 men and 7 women, with mean age of 29.2 years and standard deviation of 16.6; mean time of profound hearing loss of 70.3 months, with standard deviation of 82.5 and mean time of CI use of 46.3 months and standard deviation of 33.9. As far as etiology is concerned, 36% had hearing loss as a consequence of meningitis, 29% was idiopathic, 7% hereditary, 7% caused by encephalitis and 21% caused by head injury.
• Control group: matched the experimental group as far as gender and age are concerned. We assessed 14 individuals with normal hearing and without a history that could suggest auditory processing alteration, seven females and seven males, with mean age of 29.7 years and standard deviation of 16.1.

Since this is a preliminary study, we did not consider for result analysis purposes the following variables: type of CI, speech codifying strategy and external device.

## PROCEDURES

Individuals from the experimental group were evaluated in an open field with the speaker positioned at 0º azimuth and 60 cm away from the individual. The CI was on, the external component controls were positioned in the most common settings before the new speech processor programming took place.

For the control group, the tests were carried out under two situations: with supra-aural phones and in the open field, with the aim of analyzing the test applicability in the open field and characterize the normality.

### Temporal ordering tests

We used the 1998 version of the Audiology Illustrated stored in the LCC - Central Auditory Tests CD20. To perform the PPST and the DPST we used 6 sequences during the training phase, 40 in the open field and 60 sequences with the supra-aural phones, 30 for the right ear and 30 for the left. The test was applied in the intensity of 50dBSL, and as a reference we used the auditory threshold mean values in the frequencies of 500, 1000 and 2000Hz obtained from the threshold tonal audiometry and by means of open field audiometry^21^, ^22^.

For the PPST we used tone sequences with the same duration and with variable frequencies: low frequency tone of 880Hz and high frequency of 1122Hz. The tones were combined in 6 different patterns: high-high-low (AAG), high-low-low (AGG), high-low-high (AGA), low-low-high (GGA), low-high-low (GAG) and low-high-high (GAA). The sequences were introduced randomly^21^.

DPST was made up of stimuli that differed in terms of duration, fixed frequency, and they could be long -L (500ms) or short-C (250ms). As it happened in the PPST, the tones were combined in 6 different patterns: LLC, LCL, LCC, CLL, CLC, CCL^22^.

The individuals were required to verbally answer the patterns they had heard, and such answers were written down by the evaluator in a printed recording sheet. For result analyses, we calculated the number of correct answers, inversions and errors and the percentage established. The inversions were considered errors.

We used descriptive statistics (mean, median, standard deviation, minimum and maximum) in order to characterize the series in terms of age, time of CI use and sensorial deprivation time, and to introduce the rates of PPST and DPST.

Wilcoxon”s non-parametric test was applied with the aim of comparing the results between temporal ordering tests. In order to compare the results obtained in the open field and with supra-aural phones, we used the Friedman”s non-parametric test. The influence of the gender variable in the results of the experimental and control groups was analyzed by the Mann-Whitney U non-parametric test. Spearman”s non-parametric test was used to check the correlation between age and test performance, as well as the percentages of correct answers in the PPST and DPST for both groups evaluated (experimental and control). The level of significance used for all the tests was 0.05.

## RESULTS

Control group individuals” temporal ordering test result analyses, by means of the Mann-Whitney U non-parametric test did not show statistically significant difference between men and women for PPST and DPST, p=0.06 and p=0.25, respectively. Thus, in the analysis carried out after that, the gender variable was not considered among the 14 individuals in the control group.

On Table 1 we see the summary of the mean, median, standard deviation, minimum and maximum values obtained from the temporal ordering tests (PPST and DPST), with a supra-aural phone on the right ear, left ear and open field for control individuals, as well as the results from the Friedman”s test comparing the assessment situation.

**Table 1:** Mean, median, standard deviation and minimum and maximum values of the percentage of correct answers in the temporal ordering tests (frequency and duration) applied to control group individuals. Friedman test to compare the evaluation situations.

TEMPORAL ORDERING TEST						
		Frequency pattern			Duration pattern	
	Right side supra-aural phone	Left side supra-aural phone	Open field	Right side supra-aural phone	Left side supra-aural phone	Open field
Mean (%)	64,6	62,3	63,4	62,5	58,0	64,6
sd (%)	28,7	31,1	28,8	23,0	27,0	23,9
Median (%)	71,5	77,0	70,0	57,0	57,0	62,5
Minimum (%)	13,0	7,0	12,5	30,0	14,0	17,5
Maximum (%)	100	100	97,5	100	100	100

P		0,87			0,11	

Legend: sd standard deviation

P<0.05 - significant

We did not see statistically significant differences between the evaluations carried out for PPST and DPST with the supra-aural phone on the right ear, left ear and in the open field, p=0.87 and p=0.11, respectively.

Comparing PPST and DPST performances with the Wilcoxon non-parametric test, we did not observe statistically significant difference (p=0.36); however, there was a positive and statistically significant Spearman”s correlation, rs=0.79.

Comparative analyses between the control and experimental groups

Table 2 shows the mean, median, standard deviation, minimum and maximum values associated with the results obtained in the temporal ordering test evaluations in the open field for both groups, as well as the result from the Mann-Whitney U test to compare the performance between the two groups.

**Table 2:** Mean, median, standard deviation, minimum and maximum value of correct answers in the temporal ordering tests (frequency and duration) applied to the control and experimental groups in the open field assessment. Mann-Whitney U test to compare the two groups.

TEMPORAL ORDERING TEST				
	Padrão de freqüência		Padrão de duração	
	Control Group	Experimental Group	Control Group	Experimental Group
Mean (%)	63,4	48,7	64,6	59,6
sd (%)	70,0	38,7	62,5	56,2
Median (%)	28,8	27,7	23,9	25,8
Minimum (%)	12,5	17,5	17,5	10,0
Maximum (%)	97,5	95,0	100	97,5

P	0,29		0,52	

Legend: sd standard deviation

P<0.05 - significant

The non-parametric Mann-Whitney U test did not show statistically significant difference between the score achieved by the control and experimental group individuals in PPST and DPST, with p=0.29 and p=0.52, respectively.

**Figure 1:**
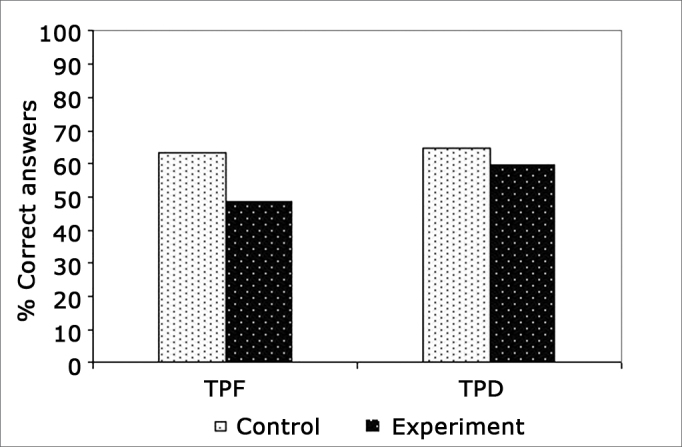
Average correct answers percentage in the frequency patterns test and in the duration patterns test in the open field of control and experimental group individuals.

Figure 1 represents the percentage of correct answers in PPST and DPST in the open field for individuals from the control group when compared to individuals from the experimental group.

When the performances were compared using the Wilcoxon non-parametric test there was not significant difference between the PPST and DPST performances in the experimental group (p=0.55).

Temporal ordering tests showed a positive correlation (rs=+0.36) with the use of Spearman”s correlation, showing that when there is a weak trend towards the score in one test it would increase together with the score of another test.

## DISCUSSION

Speech perception involves a complex interaction between factors that go from a simple detection, identification, categorization and recognition of acoustic signs, including the discrimination of the different spectra, duration of temporal characteristics, sequential forms and speech sound rhythm. The processing of speech sound acoustic hints depends on the proper perception of frequency spectrum and stimulus duration as a sequence of events^12^, ^21^.

Auditory processing evaluation by means of behavioral tests in the post-surgical routine of CI programs may provide important subsidies that will help in the analyses of individual performance in relation to speech perception, having seen that other variables must be considered when results obtained with CI are discussed, such as the hearing impairment etiology, the type of electrode insertion, the CI model, speech codifying strategies, amongst others.

The task of assessing the auditory processing skill wholeness is very complex, especially in those individuals with hearing loss with alterations in sound sensation and perception distortion caused by the cochlear disorder^10^. Thus, when there is an alteration in sound perception, all the subsequent mechanisms will be altered, making it difficult to process acoustic information^9^.

Notwithstanding, performance in temporal ordering tests does not alter significantly because of cochlear distortions after sound amplification and, possibly, because these are tests with non-verbal stimuli to assess auditory processing without the participation of language processes^8^, ^12^, ^13^, ^21^.

Thus, in analyzing the results obtained in the tests of duration and frequency of sequential patterns used in CI users, the implanted device must be considered an integral part of the entire set of structures responsible for auditory processing.

In the temporal ordering tests carried out in the open field with normal hearing individuals, the gender variable did not influence the results, being in agreement with other studies carried out with individuals between 7 and 16 years of age^7^, ^8^.

According to Table 1, we did not see significant differences among the results obtained from the ears separately and the results obtained in the open field for the temporal ordering tests. Such findings corroborate reports in the literature that state that both PPST and DPST can be carried out in the open field^5^, ^6^.

On Table 2 and on Figure 1 we find the results obtained from the temporal ordering tests for the control and experimental groups performed in the open field. We did not see a statistically significant difference in the performance of these standard tests of duration and frequency between the two groups, as well as between the tests, both for the control and the experimental groups. The findings show that the CI provides a hearing sensation that is enough to obtain a good performance in the tasks that require temporal ordering skills, and there is no difference as to the level of difficulty in perceiving the sound characteristics associated with frequency and duration. The electrical stimuli provided by the CI are accurate in the control of the auditory nerve firings, making the CI effectively represent the temporal information of the sound stimulus^16^, ^17^.

There are a number of investigations ongoing with the aim of checking the influence of the codifying strategies and the type of CI used in the performance in these behavioral tests of auditory evaluation.

## CONCLUSION

The results obtained lead us to conclude that individuals users of CI evaluated in this study presented similar performance in the temporal ordering test (frequency and duration patterns) when compared to the group of individuals with normal hearing.
